# Validation of Quantitative Light-Induced Fluorescence Digital Analysis for Assessing Early Dental Caries Depth: A Micro-Computed Tomography-Based *In Vitro* Study

**DOI:** 10.3390/dj13030090

**Published:** 2025-02-20

**Authors:** Ji-Hyun Min

**Affiliations:** Department of Dental Hygiene, College of Health and Medical Sciences, Cheongju University, 298 Daesung-ro, Cheongwon-gu, Cheongju 28503, Chungcheongbuk-do, Republic of Korea; jhminl@cju.ac.kr or jhmin@cju.ac.kr; Tel.: +82-43-229-8375

**Keywords:** dental caries, early dental caries, lesion depth, quantitative light-induced fluorescence, X-ray microtomography

## Abstract

**Background/Objectives:** This study aimed to evaluate the analytical validity of micro-computed tomography (µCT) and compare it with quantitative light-induced fluorescence digital (QLFD) for assessing early dental caries (EDC) lesion depth (LD). This study was conducted in a destructive *in vitro* setting. **Methods:** EDC lesions were artificially induced in 28 bovine tooth specimens for 10, 20, 30, 40, 50, 60, and 70 days, respectively. The lesions were analyzed using µCT and QLFD, with grayscale intensity thresholds set at 90% and 95%. A Pearson correlation analysis was performed to evaluate the relationship between lesion depths measured using micro-computed tomography (LD_µCT_) and quantitative light-induced fluorescence digital (LD_QLFD_) across different demineralization periods. Additionally, a Bland–Altman analysis was conducted to assess agreement between the two methods and identify systematic differences and potential bias. A regression analysis was further conducted with LD_QLFD_ as the independent variable and LD_µCT_ as the dependent variable. **Results:** LD_µCT_ was consistently lower than LD_QLFD_ across all demineralization periods. The Pearson correlation analysis demonstrated a strong correlation between LD_µCT_ and LD_QLFD_ (r = 0.898–0.977, *p* < 0.001). The Bland–Altman analysis demonstrated a high level of agreement, with most data points falling within the 95% limit of agreement. Furthermore, the regression analysis suggested that QLFD may serve as a reliable complementary tool for lesion depth assessment. **Conclusions:** Although µCT and QLFD operate based on different principles, the findings of this study, along with the cost-effectiveness and ease of use of QLFD, suggest that QLFD may serve as a complementary tool to µCT in experimental research.

## 1. Introduction

Early dental caries (EDC) is a critical stage of tooth decay where mineral loss occurs without structural breakdown. During this stage, remineralization can restore the tooth to its normal state, making it a golden opportunity for intervention [1]. Once EDC progresses to structural collapse and cavity formation, restoration becomes impossible, highlighting the importance of early management and treatment. To achieve this, diverse and in-depth foundational studies on EDC are essential.

The gold standard for the *in vitro* evaluation of mineral loss and lesion depth in EDC is transverse microradiography (TMR) [2,3]. TMR allows for a precise analysis of lesion depth and integrated mineral loss (ΔZ, volume % mineral × µm) in transverse sections of EDC. However, TMR requires a preprocessing step to adjust the tooth specimen to a thickness of 100 µm, and the overall analysis process is complex. Additionally, the equipment is expensive, limiting its widespread use worldwide. As an alternative, micro-computed tomography (µCT) has been proposed. A previous study has demonstrated significant correlations between TMR and µCT in assessing enamel caries lesions, suggesting that µCT can serve as a viable substitute [4]. Additionally, unlike TMR, µCT has the advantage of not requiring the specimens to be preprocessed to a thickness of 100 µm. Nevertheless, µCT remains an expensive tool that is not easily accessible to individual researchers.

Various tools have been developed for the non-invasive clinical detection, diagnosis, severity assessment, and progression analysis of EDC [5,6,7,8]. Among them, the clinical applicability and analytical validity of quantitative light-induced fluorescence (QLF, Inspektor Research Systems BV, Amsterdam, Netherlands) have been well established in various studies [9,10,11]. QLF quantifies the depth of EDC based on fluorescence loss (delta F, ΔF) values. In hydroxyapatite (Ca_10_(PO_4_)_6_(OH)_2_), mineral loss increases porosity, leading to enhanced light scattering and reduced fluorescence intensity compared to sound enamel [12]. Several studies have demonstrated a strong correlation between ΔF values and the extent of mineral loss in EDC [9,10,11,12]. Currently, QLF is widely used in dental schools, clinics, and research institutions, with a growing number of related studies and increasing QLF-related publications indexed in PubMed [13,14].

QLF is a non-destructive method for evaluating EDC. However, unlike previous studies, this study aimed to analyze EDC in an *in vitro* destructive analysis setting, similar to the analytical methods of TMR and µCT, which involve sectioning tooth specimens or sacrificing research subjects, and to evaluate its validity. To achieve this, artificially induced EDC lesions in transverse sections were analyzed and compared using QLF and µCT. The objective of this study was to verify the reliability and validity of transverse section analysis of EDC using QLF. This study hypothesizes that a QLF analysis is a valid method for assessing the depth of EDC in transverse sections and that it strongly correlates with µCT.

## 2. Materials and Methods

### 2.1. Preparation and Fabrication of Specimens

A single specimen was prepared from each sound bovine permanent anterior tooth, which exhibited no visible signs of demineralization, cracks, or structural defects. Teeth presenting fractures, morphological abnormalities, or any evidence of prior mineral loss were excluded. Each selected tooth was sectioned into a standardized 5 mm × 3 mm specimen using a diamond disc bur (NTI-Kahla GmbH, Kahla, Germany). The sectioned teeth were embedded in acrylic resin (curing acrylic denture repair material; Vertex, Soesterberg, the Netherlands) molds, with the enamel surface of the labial side exposed. The specimens were sequentially ground and polished using silicon carbide paper (400p to 2400p; Allied High Tech Products Inc., Rancho Dominguez, CA, USA) mounted on a polishing machine (RB 209 Minipol, R&B Inc., Daejeon, Korea). To prevent interference with QLFD imaging, transparent nail varnish without fluorescent components was applied to a 1 mm × 3 mm area on one side of the specimen.

**Figure 1:**
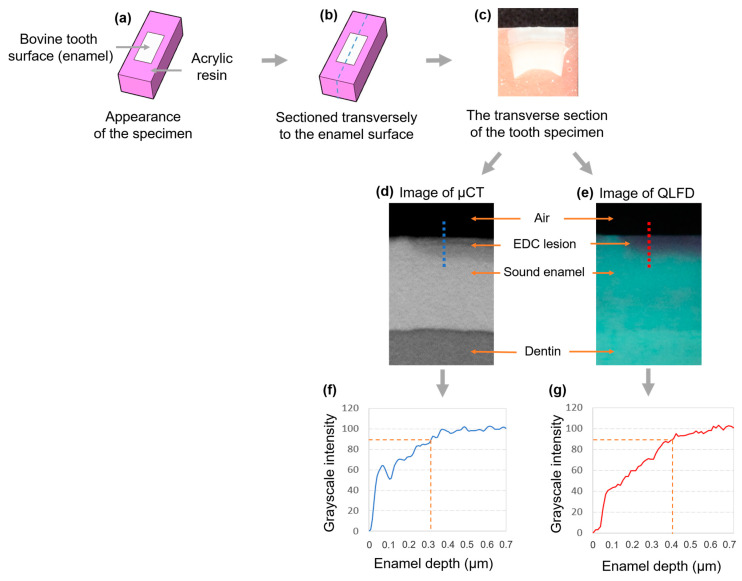
A scheme of this study. The specimens were prepared by embedding them in acrylic resin, exposing the enamel surface of the bovine tooth (**a**). Subsequently, the specimens were transversely sectioned along the enamel surface (**b**). µCT images, as shown in (**d**), and QLFD images, as shown in (**e**), were obtained from transverse sections of the tooth specimen, such as (**c**). In the µCT and QLFD images, the dashed lines indicate the regions where grayscale intensity was measured. The grayscale intensity profile was plotted along the path starting from the air portion (completely black), passing through the enamel surface and the EDC lesion, and reaching the sound enamel region. The lesion depth was assessed based on the grayscale intensity thresholds of 90% and 95% in the grayscale intensity profile (**f**,**g**). Additionally, the blue dashed and solid lines represent µCT, while the red dashed and solid lines correspond to QLFD.

A solution for inducing artificial caries was prepared by adding tribasic calcium phosphate (Ca_5_(OH)(PO_4_)_3_; C5267, Sigma Aldrich, St. Louis, MO, USA) to a 1M lactic acid solution (CH_3_CH(OH)COOH; 69785, Sigma Aldrich, St. Louis, MO, USA). Hydrochloric acid (HCl; 07102, Sigma Aldrich, St. Louis, MO, USA) was then added for 30 min to maintain a pH of 4.8, creating a saturated solution. Subsequently, 2% Carbopol (Carbopol ETD 2050 polymer; The Lubrizol Corporation, Wickliffe, OH, USA) was added, and the final pH was adjusted to 4.8 [15]. The sample size was determined using G*Power software (version 3.1.9.4) for a one-way ANOVA. With an effect size of 0.25, a Type I error rate (α) of 0.05, a statistical power of 0.7, and 7 groups, the total calculated sample size was 189 specimens. A minimum of 27 specimens were required per group, and in this study, 28 specimens were allocated to each group. Seven specimens were placed in a 50 mL demineralization solution and incubated at 37 °C. Demineralization was performed for 10, 20, 30, 40, 50, 60, and 70 days, with the solution being replaced every 20 days [16]. Using a low-speed saw (Minitome, Struers, Copenhagen, Denmark), the specimens were sectioned perpendicular to the enamel surface. To prevent fracturing of the enamel surface, which was expected to be weakened due to mineral loss, sectioning was performed at 200 rpm [7]. Figure 1 presents the scheme of this study, illustrating the experimental workflow and analysis process.

### 2.2. Micro-Computed Tomography (µCT) Analysis

µCT images (SkyScan 1273, Bruker, Karlsruhe, Germany) were acquired using X-rays generated at 100 kV and 80 µA. A 1 mm aluminum filter and a 0.038 mm copper filter were applied to reduce beam hardening artifacts and enhance image quality in µCT imaging. To minimize artifacts and noise and enhance data accuracy, ring artifact correction, flat field correction, frame averaging, and random movement were applied. Each tooth specimen was scanned within a rotation range of 206.25°, and the total scan time was 4 min and 46 s. The distance from the X-ray source to the sample was 53.545 mm, while the distance from the sample to the detector (camera) was 500.742 mm. The data were saved as 1375 TIFF files, with each file having a resolution of 3072 × 1944 pixels. The reconstructed 3D images had an isotropic voxel size of 7.999 µm. Cross-sectional surface images of the tooth specimens with appropriate grayscale intensity and contrast were obtained using dedicated software (3D.SUITE, version 1.7.0.0, Bruker, Karlsruhe, Germany).

### 2.3. Quantitative Light-Induced Fluorescence Digital (QLFD) Analysis

Cross-sectional surface images were captured using QLF-digital (QLFD, 2+Biluminator™; Inspektor Research Systems BV, Amsterdam, the Netherlands) while blocking all external light to minimize ambient light interference, and a black background was used to optimize contrast. The blue light imaging conditions were set to ISO 1600, a shutter speed of 1/20 s, and an aperture value of 8.0.

### 2.4. The Generation of a Standardized Grayscale Intensity Profile

Image analysis was performed using ImageJ software (version 1.54k, National Institutes of Health, Bethesda, MD, USA). Grayscale intensity profiles were generated across the enamel region, starting from the outer region (air), passing through the enamel surface, demineralized EDC regions, and extending into sound enamel. The transition point at the beginning of the µCT and QLF profiles represents the shift from air to the enamel surface, marking the outermost boundary of the specimen. The region where grayscale intensity was present was identified as the superficial enamel lesion. Identical regions of interest were selected and analyzed in both µCT and QLFD images to ensure consistency. To standardize the profiles, the grayscale intensity of the black background was set to 0, while the maximum brightness in the sound enamel region was normalized to 100. These values were used to construct standardized intensity profiles, where grayscale intensity was plotted on the vertical axis and enamel depth on the horizontal axis, with the tooth surface at 0 depth as the reference baseline. Consequently, the enamel depths corresponding to grayscale intensity thresholds of 90% and 95% were measured in both µCT and QLFD images [4,7,16]. The image analysis of the grayscale intensity profile was performed by a single researcher.

### 2.5. Statistical Analysis

A paired *t*-test was conducted to compare the mean enamel lesion depths (LDs) corresponding to 90% grayscale intensity values obtained from µCT (90% LD_µCT_) and QLFD (90% ED_QLFD_). Differences in the mean values of 90% LD_µCT_ and 90% LD_QLFD_ across demineralization periods were analyzed using one-way ANOVA followed by Tukey’s post hoc test. The same analyses were performed for the enamel depths corresponding to 95% grayscale intensity values obtained from µCT (95% LD_µCT_) and QLFD (95% LD_QLFD_). The correlations among 90% LD_µCT_, 90% LD_QLFD_, 95% LD_µCT_, and 95% LD_QLFD_ were evaluated using Pearson correlation analysis. A simple linear regression analysis was performed with LD_QLFD_ as the independent variable and LD_µCT_ as the dependent variable. Additionally, Bland–Altman analysis was conducted to assess the agreement between LD_QLFD_ and LD_µCT_ at both the 90% and 95% thresholds, with the mean difference and 95% limits of agreement (LOAs) visualized in Bland–Altman plots. All statistical analyses were conducted using IBM SPSS Statistics version 29.0 (IBM Corp., Armonk, NY, USA), and a *p*-value of less than 0.05 was considered statistically significant.

## 3. Results

### 3.1. Comparison of Enamel Lesion Depth at 90% and 95% Grayscale Intensity Thresholds

As the demineralization period increased from 10 to 70 days, both 90% LD_µCT_ and 90% LD_QLFD_ showed a significant increase (*p* < 0.001). Across all demineralization periods, 90% LD_µCT_ consistently exhibited lower values than 90% LD_QLFD_. After 10 days of demineralization, 90% LD_µCT_ was 175.98 ± 52.20 µm, while 90% LD_QLFD_ was 248.57 ± 53.72 µm. After 70 days, 90% LD_µCT_ increased to 336.53 ± 72.07 µm, and 90% LD_QLFD_ increased to 443.18 ± 86.87 µm (Table 1).

Both 95% LD_µCT_ and 95% LD_QLFD_ significantly increased as the demineralization period lengthened (*p* < 0.001). Across all demineralization periods, 95% LD_µCT_ consistently showed lower values than 95% LD_QLFD_. For instance, after 10 days of demineralization, 95% LD_µCT_ was 202.55 ± 52.88 µm, while 95% LD_QLFD_ was 280.00 ± 57.46 µm. After 70 days, 95% LD_µCT_ increased to 377.67 ± 80.12 µm, and 95% LD_QLFD_ increased to 473.18 ± 86.47 µm (Table 2).

### 3.2. Correlation Between LD_µCT_ and LD_QLFD_

Strong positive correlations were observed between LD_µCT_ and LD_QLFD_ at both 90% and 95% grayscale intensity values (r = 0.898–0.977, *p* < 0.001). The correlation was slightly higher between 90% LD_µCT_ and 90% LD_QLFD_ (r = 0.920) compared to 95% LD_µCT_ and 95% LD_QLFD_ (r = 0.901, Table 3).

### 3.3. Linear Regression Analysis

At the 90% grayscale intensity threshold, the regression model showed an R^2^ value of 0.847, indicating that 90% LD_QLFD_ had a strong influence on 90% LD_µCT_. The regression equation was Y = 0.819X − 21.857 (where Y represents 90% LD_µCT_ and X represents 90% LD_QLFD_). The Durbin–Watson value was 1.993, indicating no autocorrelation and the independence of residuals. The model’s goodness of fit was significant, with an F-value of 1075.323 and a *p*-value < 0.001, demonstrating that the regression equation was statistically significant. Similar results were observed at the 95% grayscale intensity threshold. The R^2^ value was 0.811, and the regression equation was calculated as Y = 0.820X − 16.447. Both models yielded statistically significant results (*p* < 0.001). The Durbin–Watson value was 1.840, and the model’s goodness of fit was significant, with an F-value of 833.050 and a *p*-value < 0.001, confirming the statistical significance of the regression equation (Table 4).

### 3.4. Bland–Altman Plot

**Figure 2:**
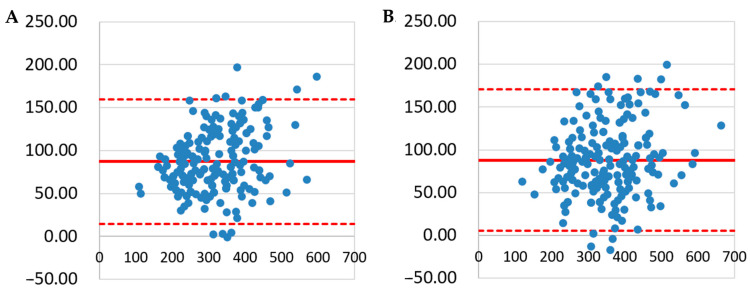
A Bland–Altman analysis of LD_µCT_ and LD_QLFD_ measurements. (**A**) A Bland–Altman plot of LD_µCT_ and LD_QLFD_ at the 90% threshold. (**B**) A Bland–Altman plot of LD_µCT_ and LD_QLFD_ at the 95% threshold. The solid red line represents the mean difference between the two methods, while the dashed red lines indicate the 95% limits of agreement (LOA). In (**A**,**B**), the majority of data points fall within the LOA, confirming a high level of agreement between LD_µCT_ and LD_QLFD_ at both the 90% and 95% thresholds.

The Bland–Altman analysis demonstrated a high level of agreement between LD_µCT_ and LD_QLFD_ at both the 90% and 95% grayscale intensity thresholds. The majority of data points were distributed within the 95% LOA, indicating that the differences between the two methods remained within an acceptable range. The mean difference (bias) between LD_µCT_ and LD_QLFD_ was relatively small, and no systematic trend was observed across the measurement range (Figure 2).

## 4. Discussion

This study aimed to test the hypothesis that QLFD is a valid method for assessing the depth of EDC in transverse sections and that it exhibits a strong correlation with µCT. The findings support this hypothesis, as QLFD demonstrated a significant correlation with µCT in measuring lesion depth. These results indicate that QLFD may serve as a practical alternative to µCT for laboratory-based studies on EDC. Notably, the analytical approach employed in this study differs from previous research in that it specifically focuses on the transverse sections of teeth.

To assess the maximum depth of EDC, artificially formed on the enamel and extending to areas considered sound enamel, 90% and 95% thresholds were established, as referenced in previous studies [17,18]. Significant correlations were observed between µCT and QLFD at both thresholds (r = 0.898–0.977, *p* < 0.001). Furthermore, a significant regression equation was identified between 90% LD_QLFD_ and 90% LD_µCT_, indicating that for every unit increase in 90% LD_QLFD_, 90% LD_µCT_ decreased by 0.819. Similarly, for 95% LD_QLFD_, a unit increase corresponded to a decrease of 0.820 in 95% LD_µCT_. In both cases, the standardized beta coefficients were 0.920 and 0.901, respectively, indicating a strong influence of the independent variable, QLFD, on µCT measurements. These findings validate the effectiveness of QLFD in assessing mineral loss in transverse sections of EDC.

The grayscale intensity thresholds of 90% and 95% were selected based on their validated application in lesion depth assessment studies using µCT and TMR. Hamba et al. [4] demonstrated that the lesion depth, defined as the depth at which the mineral content reaches 95% of the maximum mineral density, strongly correlates with TMR measurements (r > 0.86, *p* < 0.001). A 100% grayscale threshold was not employed due to potential imaging artifacts, including beam-hardening effects in µCT, which may lead to an underestimation of the lesion depth. In this study, both the 95% and 90% thresholds were evaluated, and the results show a strong correlation with the µCT measurements. Furthermore, the Bland–Altman analysis confirmed a high level of agreement between the lesion depth values obtained using the 90% and 95% thresholds, supporting their consistency with the µCT measurements.

Most previous studies on QLFD have focused on validating its utility as a clinical research tool for surface-level analysis of EDC [5,9,10,11,12]. In earlier research utilizing QLF, mineral loss in early caries lesions was assessed, and both artificial and natural lesions were reported to exhibit a linear relationship between fluorescence loss and mineral loss [12]. The slope of this relationship indicated that a 10% fluorescence loss corresponded to a mineral loss of 0.15 kg·m^−2^ [12]. Other studies evaluated the sensitivity and specificity of QLFD for caries detection, demonstrating that fluorescence loss data accurately reflect the lesion depth and mineral status [11]. Additionally, numerous studies have reported that QLFD analysis of the tooth surface reflects the depth of EDC [10,19,20,21]. Although prior QLFD studies primarily focused on surface-level analysis, the linear relationship identified in this study between LD_µCT_ and LD_QLFD_ in transverse sections is consistent with previous findings.

µCT is a high-resolution imaging tool capable of precisely evaluating early caries lesion depth in three dimensions. However, its high cost and time-intensive analysis limit its broader application, particularly in experimental and clinical settings [9]. Although QLFD is generally considered a non-destructive imaging technique, its application in this study required a destructive approach, as transverse sectioning of specimens was performed to obtain LD_QLFD_ measurements [14]. QLFD consistently measured greater LDs than µCT across all demineralization periods. µCT quantifies mineral density based on X-ray attenuation, whereas QLF detects demineralization by measuring fluorescence loss, which correlates with mineral loss. Despite the strong correlation between the two methods, methodological differences between µCT and QLFD suggest that LD_QLFD_ should be considered a complementary approach rather than a direct substitute for LD_µCT_.

Additionally, TMR remains the gold standard for mineral loss assessment but is limited by its high cost, time-consuming nature, and need for thin-sectioned specimens [9]. The observed correlation between LD_µCT_ and LD_QLFD_ suggests that QLFD may serve as a complementary tool for lesion depth evaluation. Future studies should further investigate its relationship with both LD_µCT_ and TMR to refine its application in caries research. This study assessed the analytical validity of QLFD in a destructive *in vitro* setting using standardized artificial EDC lesions. While this study does not aim to replace in vivo research, future studies should investigate the applicability of QLF in extracted human teeth, considering various tooth types and lesion locations.

Despite the destructive nature of the transverse sectioning approach, QLFD demonstrated strong potential for early caries lesion assessment. Given its cost-effectiveness and objective fluorescence-based quantification, QLFD is expected to be valuable for experimental research applications.

## 5. Conclusions

This study demonstrated that QLFD can serve as a valuable tool for the quantitative assessment of EDC in laboratory settings, complementing µCT in evaluating lesion depth and progression. Despite the destructive nature of the transverse sectioning approach used in this study, QLFD exhibited strong potential for mineral loss assessment, supporting its applicability in experimental research.

## Figures and Tables

**Table 1:** Enamel depth at 90% grayscale intensity (unit: µm).

Demineralization Period	N	Mean ± S.D.		*p*-Value ^2^
		90% LD_µCT_^1^	90% LD_QLFD_^1^	
10 days	28	175.98 ± 52.20 ^a^	248.57 ± 53.72 ^a^	<0.001
20 days	28	226.26 ± 66.21 ^ab^	302.71 ± 74.81 ^ab^	<0.001
30 days	28	275.11 ± 69.91 ^bc^	357.75 ± 72.27 ^bc^	<0.001
40 days	28	283.96 ± 75.56 ^de^	361.71 ± 76.54 ^d^	<0.001
50 days	28	291.96 ± 41.62 ^de^	393.86 ± 47.54 ^de^	<0.001
60 days	28	332.53 ± 78.81 ^e^	425.18 ± 78.93 ^e^	<0.001
70 days	28	336.53 ± 72.07 ^e^	443.18 ± 86.87 ^e^	<0.001
*p*-value ^3^		<0.001	<0.001	

^1^ Different letters (a–e) indicate statistically significant differences as determined by Tukey’s post hoc test. ^2^ Statistical significance was determined using paired *t*-tests (*p* < 0.05). ^3^ Statistical significance was determined using one-way ANOVA (*p* < 0.05).

**Table 2:** Enamel depth at 95% grayscale intensity (unit: µm).

Demineralization Period	N	Mean ± S.D.		*p*-Value ^2^
		95% LD_µCT_^1^	95% LD_QLFD_^1^	
10 days	28	202.55 ± 52.88 ^a^	280.00 ± 57.46 ^a^	<0.001
20 days	28	255.68 ± 67.82 ^ab^	333.75 ± 77.80 ^a^	<0.001
30 days	28	309.46 ± 78.14 ^bc^	396.46 ± 85.66 ^b^	<0.001
40 days	28	321.10 ± 73.14 ^c^	397.79 ± 66.85 ^b^	<0.001
50 days	28	330.24 ± 46.43 ^cd^	431.04 ± 51.21 ^bc^	<0.001
60 days	28	362.81 ± 75.11 ^cd^	463.39 ± 81.07 ^c^	<0.001
70 days	28	377.67 ± 80.12 ^d^	473.18 ± 86.47 ^c^	<0.001
*p*-value ^3^		<0.001	<0.001	

^1^ Different letters (a–d) indicate statistically significant differences as determined by Tukey’s post hoc test. ^2^ Statistical significance was determined using paired *t*-tests (*p* < 0.05). ^3^ Statistical significance was determined using one-way ANOVA (*p* < 0.05).

**Table 3:** Correlation between QLF-D and µCT.

	90% LD_µCT_	90% LD_QLFD_	95% LD_µCT_
90% LD_QLFD_	0.920 (*p* <0.001)		
95% LD_µCT_	0.946 (*p* <0.001)	0.911 (*p* <0.001)	
95% LD_QLFD_	0.898 (*p* <0.001)	0.977 (*p* <0.001)	0.901 (*p* <0.001)

The Pearson correlation coefficients and *p*-values were obtained using a two-tailed test.

**Table 4:** Linear regression analysis results.

Grayscale Intensity Threshold	Constant (Intercept)	Coefficient (B)	Standardized Beta Coefficient	R^2^ Value	F	*p*-Value
0.90	−21.857	0.819	0.920	0.7970.847	1075.323	<0.001
0.95	−16.447	0.820	0.901	0.7200.811	833.050	<0.001

Regression analysis was conducted to evaluate the relationship between lesion depth measured by µCT and QLFD.

## Data Availability

The original data presented in the study are openly available at https://doi.org/10.5281/zenodo.14858632.

## Abbreviations

The following abbreviations are used in this manuscript:

EDC	Early dental caries
TMR	Transverse microradiography
QLF	Quantitative light-induced fluorescence
QLFD	Quantitative light-induced fluorescence-digital
µCT	Micro-computed tomography
LD	Lesion depth
90% LD_µCT_	Lesion depth at 90% grayscale intensity values obtained from µCT
90% LD_QLFD_	Lesion depth at 90% grayscale intensity values obtained from QLFD
95% LD_µCT_	Lesion depth at 95% grayscale intensity values obtained from µCT
95% LD_QLFD_	Lesion depth at 95% grayscale intensity values obtained from QLFD
